# Controlling Nutritional Status (CONUT) as a Novel Postoperative Prognostic Marker in Breast Cancer Patients: A Retrospective Study

**DOI:** 10.1155/2022/3254581

**Published:** 2022-12-09

**Authors:** Mengliu Zhu, Li Chen, Xiangyi Kong, Xiangyu Wang, Yingpeng Ren, Qiang Liu, Xingrui Li, Yi Fang, Jing Wang

**Affiliations:** ^1^Department of Breast Surgical Oncology, National Cancer Center/National Clinical Research Center for Cancer/Cancer Hospital, Chinese Academy of Medical Sciences and Peking Union Medical College, Beijing 100021, China; ^2^Department of Thyroid and Breast Surgery, Tongji Hospital, Tongji Medical College of Huazhong University of Science and Technology, Wuhan, Hubei 430030, China

## Abstract

**Purpose:**

This investigation seeks to elucidate the potential prognostic significance as well as the clinical utility of the controlling nutritional status (CONUT) score in breast cancer patients.

**Methods:**

Breast cancer patients managed in our center between January 2010 and December 2016 were recruited for our study. They comprised 187 patients who did not undergo neoadjuvant chemotherapy and 194 who did. A receiver operating characteristic curve (ROC) was utilized in identifying the ideal cut-off CONUT value. This cut-off score was then used to reclassify patients into those with high CONUT scores (≥1) and low CONUT scores (<1). The outcomes were analyzed by statistical methods.

**Results:**

Univariate and multivariate Cox regression survival analyses revealed that a CONUT score cut-off of 1 was able to significantly predict duration of disease-free survival (DFS) (*p* < 0.001; hazard ratio [HR]: 3.184; 95% CI: 1.786-5.677; and *p* < 0.001; HR: 2.465; 95% CI: 1.642-3.700) and overall survival (OS) (*p* < 0.001; HR: 2.326; 95% CI: 1.578-3.429; and *p* < 0.001; HR: 2.775; 95% CI: 1.791-4.300). The mean DFS and OS in those with lower CONUT scores were 41.59 (95% CI: 37.66-45.51 months) and 77.34 months (95% CI: 71.79-82.90 months), respectively. On the other hand, the average DFS and OS for all individuals in the raised CONUT score group were 39.18 (95% CI: 34.41-43.95 months) and 71.30 months (95% CI: 65.47-77.12 months), respectively. Moreover, Kaplan-Meier survival analysis revealed that those in the raised CONUT score cohort had remarkably worse DFS and OS survival rates compared to individuals in the low CONUT score cohort (Log-rank test, DFS: *χ*^2^ = 12.900, *p* = 0.0003, and OS: *χ*^2^ = 16.270, *p* < 0.0001).

**Conclusion:**

The survival times of breast cancer patients may be reliably predicted using the CONUT score. This score is an easy, convenient, readily accessible, and clinically significant means of prognosticating patients with breast cancer.

## 1. Introduction

The latest reported global cancer statistics data places breast cancer (BC) as the most frequently diagnosed malignancy around the world. BC is a notable contributor to cancer-associated morbidity and mortality in females globally, with the year 2020 yielding over 276,480 new cases and 63,220 deaths [1, 2]. The development of BC is a multistep process involving a variety of cell types, and its treatment and prevention remain challenging. Despite advances in early diagnosis and continuously improving multidisciplinary treatment modalities and protocols, the clinical course of recurrent and metastatic BC remains unsatisfactory [3, 4]. Surgery is the main treatment for BC. Key components of neoadjuvant BC treatment include chemoradiotherapy, hormonal therapy, molecular targeted therapy, and so on [5–7]. BC is a clinically heterogeneous disease, and currently available nutritional status markers have been found to be inadequate in stratifying patients according to their risk, thus limiting the extent of effective individualized treatment [8]. Treatment regimens tailored specifically to each patient will likely result in improved survival rates and prognosis. There is a need to develop more reliable biomarker panels that are able to accurately evaluate the impact of nutritional status on disease prognosis and treatment outcomes.

The latest evidence suggests an irrefutable link between nutritional status, carcinogenesis, and systemic inflammation [9]. A number of markers of systemic inflammation biomarkers such as the platelet to lymphocyte ratio (PLR), lymphocyte to monocyte (LMR), systemic immune inflammation index (SII), systemic inflammation response index (SIRI), and neutrophil to lymphocyte ratio (NLR) have been verified as independent prognostic factors for a myriad of malignancies [10–14]. Cancer cell migration, invasion, and proliferation have been found to be supported by a number of proinflammatory mediators, for example, interferon-*γ* (IFN-*γ*), interleukin-6 (IL-6), and tumor necrosis factor *α* (TNF-*α*) [15–17]. Additionally, preoperative nutritional status, including albumin, also appears to correlate to cancer prognosis [18]. In many malignancies, albumin synthesis is reduced by higher concentrations of peripheral TNF-*α* and IL-6, both of which are well-known proinflammatory mediators [19].

The Controlling Nutritional Status (CONUT) score takes into consideration both nutritional and immune status. The CONUT score is derived from three parameters total cholesterol (TC), total lymphocyte CONUT (TLC), and serum albumin concentration (ALB), and is reflective of host immune function, lipid, as well as protein metabolism [20]. The CONUT score has been shown to provide accurate prognostic and survival information in a variety of solid organ and hematological malignancies, such as gastric cancer, colorectal cancer, and multiple myeloma [21–23]. Cancer patients are often malnourished, which negatively impacts cancer progression, chemotherapy response, and postoperative outcomes. Despite these established links, the relationship between the immune system and nutritional state is not commonly explored in cancer patients. The use of the CONUT score is an immune-nutritional score with a good prognostic value that also highlights the importance of these two physiological parameters. Our investigation seeks to elaborate on the clinical utility of the CONUT score in breast cancer patients.

## 2. Methods

### 2.1. Patients

All of 381 patients treated at the Cancer Hospital Chinese Academy of Medical Sciences between January 2010 to December 2016 were retrospectively examined in this investigation. All patients had stage III histologically confirmed breast cancer and underwent a radical mastectomy. Of all, 194 cases received neoadjuvant chemotherapy, while 187 cases did not (controls). All personal data was handled in strict compliance with ethical guidelines stipulated by the 1964 Declaration of Helsinki (including its later amendments or similar ethical frameworks) and were also in line with standards upheld by the ethics committee of the Cancer Hospital Chinese Academy of Medical Sciences.

### 2.2. Eligibility Criteria and Exclusion Criteria

Eligibility criteria were: (1) patients with pathologically diagnosed with breast cancer; (2) patients who underwent primary tumor resection; (3) patients with complete clinical and follow-up medical records; (4) patients with Zubrod-ECOG-WHO (ZPS) between 0 and 2 and Karnofsky Performance Scores (KPS) ≥ 80; (5) patients with pretreatment laboratory results of peripheral blood samples (such as serum TLC, TC, and ALB).

Exclusion criteria were: (1) patients who received antitumor therapy, such as radiotherapy, chemotherapy, and targeted therapy before treatment in control group; (2) patients diagnosed with inflammatory or autoimmune diseases; (3) those found to have incomplete clinicopathological data; (4) patients who were transfused with blood products within a month prior to data collection.

### 2.3. Data Collection and Assessment

The 8th edition AJCC (American Joint Committee on Cancer) and the Union for International Cancer Control (UICC) TNM stage classification were used to assess patients' demographic and clinicopathologic parameters, which were extracted from their respective medical records [24, 25]. The following demographic parameters were collected: age, marital status, occupation, weight, height, BMI, family history, menarche age, menopause status, and so forth. The following clinicopathologic parameters were collected: hematologic parameters, imaging parameters, pathological parameters, treatment parameters, and so on. The Response Evaluation Criteria in Solid Tumors (RECIST) guidelines and Miller and Payne grade (MPG) were used to determine the degree of histological response [26, 27]. The National Cancer Institute Common Toxicity Criteria (NCI-CTC) was used to evaluate chemotherapy toxicity [28].

### 2.4. Calculation of the CONUT Score and Other Markers

Peripheral venous blood specimens were obtained within seven days before treatment in all enrolled patients. An immune-nutritional status marker, the CONUT score, was derived using TC, TLC, and ALB values. All individuals were placed into four cohorts in line with their degree of malnourishment: Normal (0-1), Light (2-4), Moderate (5-8), and Severe (9-12). The detailed information is shown in Table 1.

Prognostic nutritional index (PNI) = ALB + 5 × total lymphocyte CONUT (TLC). Systemic inflammation response index (SIRI) = neutrophil (N) × monocyte (M)/lymphocyte (TLC). Systemic Immune-inflammation Index (SII) = (neutrophil (N) × platelet (P))/lymphocyte (TLC).PLR means Platelet/Lymphocyte ratio. MLR means Monocyte/Lymphocyte ratio. NLR means Neutrophil/Lymphocyte ratio. A receiver operating characteristic curve (ROC) was utilized in identifying the ideal cut-off PNI, SIRI, SII, PLR, MLR, and NLR values.

### 2.5. Follow-Up

Follow-up was performed according to the NCCN (2020) guidelines: (1) 3-monthly for the first 1–2 years after surgery; (2) 6-monthly at 3–5 years after surgery; (3) 5-yearly until death. Disease-Free Survival (DFS) was the duration between postoperative day 1 to tumor recurrence, distant metastasis, or death from other causes. The duration between postoperative day 1 until the last follow-up or death was defined as Overall Survival (OS).

### 2.6. Statistical Analysis

GraphPad Prism Software (Version 8.0; GraphPad Inc., La Jolla, CA, USA) and SPSS 17.0 (version 17.0; SPSS Inc., Chicago, IL, USA) were used to carry out all statistical analysis. The critical optimal threshold values of related variables were identified utilizing receiver operating characteristic curves (ROC), while prognostic accuracy was evaluated using the area under the curve (AUC) value. Qualitative data was depicted in terms of the number of cases (%), with intergroup comparisons carried out via Fisher's exact test or the *χ*^2^ test. OS was determined via the Kaplan-Meier method. The survival rate between the two groups was contrasted utilizing the log-rank method. Univariate and multivariate Cox proportional hazards regression models were used to discern potential prognostic factors. Hazard ratios (HRs) and 95% confidence intervals (CIs) were used to determine the association between various parameters and breast cancer prognosis. The nomogram was conducted by the multivariate analyses, matched every prognostic variable with the corresponding score, and the sum of the scores of all potential variables is defined as the total score. The calibration curve was performed to predict the performance for DFS and OS after curative resection. And the decision curve analysis (DCA) to test predictive clinical utility. A two-tailed *p* value that was <0.05 was interpreted as achieving statistical significance.

## 3. Results

### 3.1. Patient Characteristics

Table 2 lists out all baseline clinicodemographic characteristics. ROC curve analysis highlighted 1 as the ideal cut-off CONUT score value. Participants were grouped into those having high CONUT scores (≥1) and low CONUT scores (<1). Our investigation comprised 381 patients having stage III breast cancer. Of these, 194 cases were allocated as the Treatment group (treated with neoadjuvant chemotherapy), and 187 cases were labeled as the Control group (without neoadjuvant chemotherapy and received surgery only). The median age was 50 years (range: 23-79 years). The average body mass index (BMI) of all enrolled cases was 24.95 ± 3.57. There were 152 and 229 patients in the high and low CONUT cohorts, respectively. We noted statistically significant variances with regard to preoperative body weight and tumor size between the high and low CONUT groups (*p* < 0.05). Otherwise, all parameters between the two groups were not significantly different (*p* ≥ 0.05). In control group, the results were indicated that only the weight in the preoperative high CONUT group was significantly different from those in the low CONUT group (*p* < 0.05). However, in the treatment group, we noted only the tumor size in the preoperative high CONUT group was significantly different from those in the low CONUT group (*p* < 0.05).

### 3.2. Correlation of CONUT with Blood Parameters

The ideal cut-off PNI, SIRI, SII, PLR, MLR, and NLR values are 53.50, 0.64, 434.30, 126.50, 0.19, and 1.82, respectively. Statistical analysis of the relationship between blood parameters and CONUT scores demonstrated that TC, CA153, white blood cell, hemoglobin, lymphocyte, monocyte, eosinophils, basophils, platelets, MLR, PLR, SIRI, SII, and PNI were all related to the CONUT score in all enrolled patients (*p* < 0.05). In control group, we noted that lymphocyte, MLR, PLR, SII, and PNI in the preoperative high CONUT group were significantly different from those in the low CONUT group (*p* < 0.05). In the treatment group, we noted TC, white blood cell, lymphocyte, monocyte, eosinophils, basophils, platelets, MLR, PLR, SIRI, SII, and PNI in the preoperative high CONUT group were significantly different from those in the low CONUT group (*p* < 0.05) (Table 3).

### 3.3. The Relationship between Pathological Parameters and the CONUT Score

We uncovered statistically significant variances in molecular subtypes, Ki-67 status, PR status, ER status, and TOP2A status (*p* < 0.05). And these variables were found to be significantly worse in the high CONUT group than in the low CONUT group. In control group, statistical analysis of the relationship between pathological parameters and CONUT scores demonstrated that ER status, PR status, P53 status, and TOP2A status were all related to the CONUT score (*p* < 0.05). However, there was only a molecular subtypes in the preoperative high CONUT group was significantly different from this in the low CONUT group in the treatment group (*p* < 0.05) (Table 4).

### 3.4. Disease-Free Survival and Overall Survival

Univariate analysis demonstrated that postoperative chemotherapy, postoperative radiotherapy, TC, CONUT score, and lymph vessel invasion were significant predictors for DFS; and postoperative chemotherapy, postoperative radiotherapy, TC, and CONUT score were significant predictors for OS (Table 5).

### 3.5. Associating CONUT Score with Survival Outcomes

**Figure 1:**
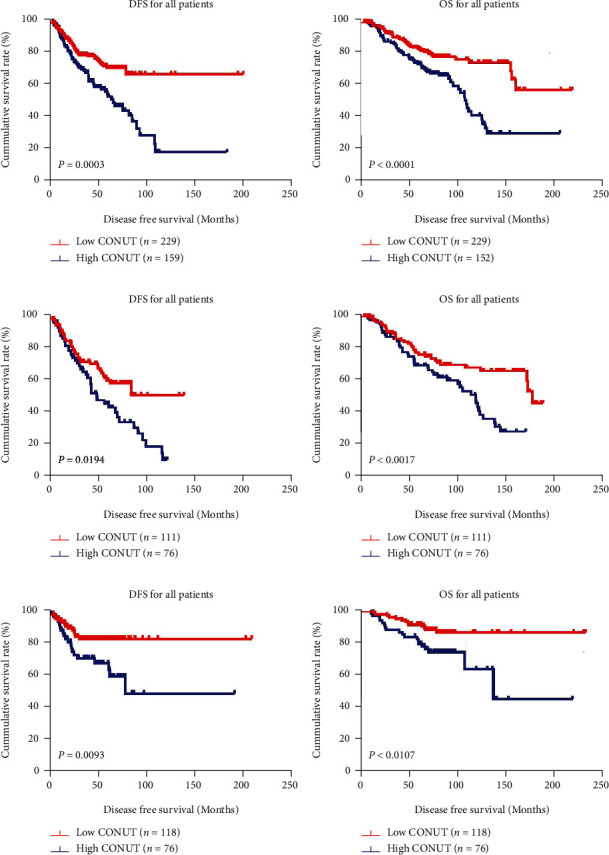
DFS and OS of patients with breast cancer. (a) Kaplan-Meier analysis of DFS for the CONUT of all patients with breast cancer. (b) Kaplan-Meier analysis of OS for the CONUT of all patients with breast cancer. (c) Kaplan-Meier analysis of DFS for the CONUT of patients with breast cancer in the Control group. (d) Kaplan-Meier analysis of OS for the CONUT of patients with breast cancer in the Control group. (e) Kaplan-Meier analysis of DFS for the CONUT of patients with breast cancer in the Treatment group. (f) Kaplan-Meier analysis of OS for the CONUT of patients with breast cancer in the Treatment group.

Both univariate and multivariate analysis revealed the CONUT score to be an independent prognosticator for DFS (*p* < 0.001; hazard ratio [HR]: 3.184; 95% CI: 1.786-5.677; and *p* < 0.001; HR: 2.465; 95% CI: 1.642-3.700, respectively) and OS (*p* < 0.001; HR: 2.326; 95% CI: 1.578-3.429; and *p* < 0.001; HR: 2.775; 95% CI: 1.791-4.300, respectively). The last follow-up time was March 10, 2021. The mean DFS for the entire population was 40.63 months (95% CI: 37.61-43.65 months), and the mean OS for the entire population was 74.93 months (95% CI: 70.87-78.99 months). The mean DFS and OS for the low CONUT score group were 41.59 months (95% CI: 37.66-45.51 months) and 77.34 months (95% CI: 71.79-82.90 months), respectively. The mean DFS and OS for the high CONUT score group were 39.18 months (95% CI: 34.41-43.95 months) and 71.30 months (95% CI: 65.47-77.12 months), respectively. Kaplan Meier survival analysis found that a higher CONUT score was predictive for worse survival (DFS and OS) in contrast to those with lower CONUT scores (Log-rank test, DFS: *χ*^2^ = 12.900, *p* = 0.0003, and OS: *χ*^2^ = 16.270, *p* < 0.0001; see in Figures 1(a) and 1(b)).

The prognostic value of the CONUT score was also evaluated in the Treatment and Control cohorts. There were 76 patients with high CONUT scores and 111 patients with low CONUT scores in the Control cohort. Kaplan Meier survival analysis revealed that patients with higher CONUT scores having significantly poorer survival outcomes in contrast to those with lower CONUT scores in terms of DFS (mean time: 39.87 months, 95% CI: 33.05-46.69 months vs. 40.63 months, 95% CI: 35.34-45.91 months) and OS (mean time: 68.90 months, 95% CI: 59.52-78.27 months vs. 75.28 months, 95% CI: 65.86-84.70 months) (Log-rank test, DFS: *χ*^2^ = 5.468, *p* = 0.0194, and OS: *χ*^2^ = 9.817, *p* = 0.0017; see in Figures 1(c) and 1(d)). In the Treatment group, there were 76 individuals with high CONUT scores and 118 individuals with low CONUT scores. Similarly, Kaplan Meier survival analysis revealed poorer outcomes in those with higher CONUT scores compared with those with lower CONUT scores in terms of DFS (mean time: 38.49 months, 95% CI: 31.66-45.31 months vs. 42.49 months, 95% CI: 36.65-48.33 months) and OS (mean time: 73.70 months, 95% CI: 66.61-80.79 months vs. 79.29 months, 95% CI: 73.03-85.55 months) (Log-rank test, DFS: *χ*^2^ = 5.468, *p* = 0.0194, and OS: *χ*^2^ = 9.817, *p* = 0.0017; see in Figures 1(e) and 1(f)).

### 3.6. Establishing CONUT-Based Nomograms in Predicting Survival Outcomes

**Figure 2:**
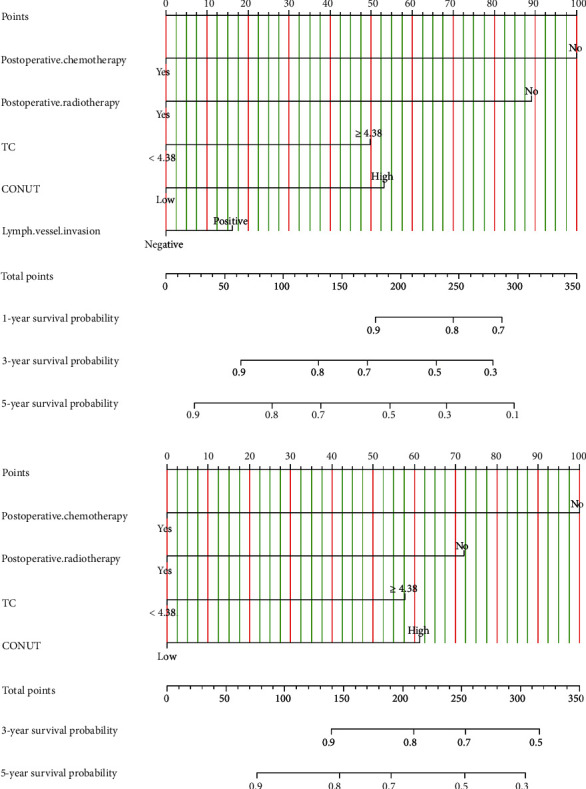
CONUT based nomogram for predicting disease-free survival (DFS) and overall survival (OS). A straight upward line is drawn to determine the points for every predictor. The sum of these points is situated on the total points axis, and a straight downward line shows the 1-year, 3-year, 5-year, and 10-year DFS and OS estimated rates. (a) CONUT based nomogram for predicting DFS; (b) CONUT based nomogram for predicting OS.

Univariate and multivariate Cox regression analyses revealed that postoperative chemotherapy, postoperative radiotherapy, TC, CONUT, and lymph vessel invasion were identified as candidate prognostic factors affecting DFS. For OS, the potential prognostic factors were postoperative chemotherapy, postoperative radiotherapy, TC, and CONUT. These prognostic factors were used to generate a DFS- and OS-predicting nomogram (Figures 2(a) and 2(b)).

### 3.7. Survival and Evaluation of CONUT Score

**Figure 3:**
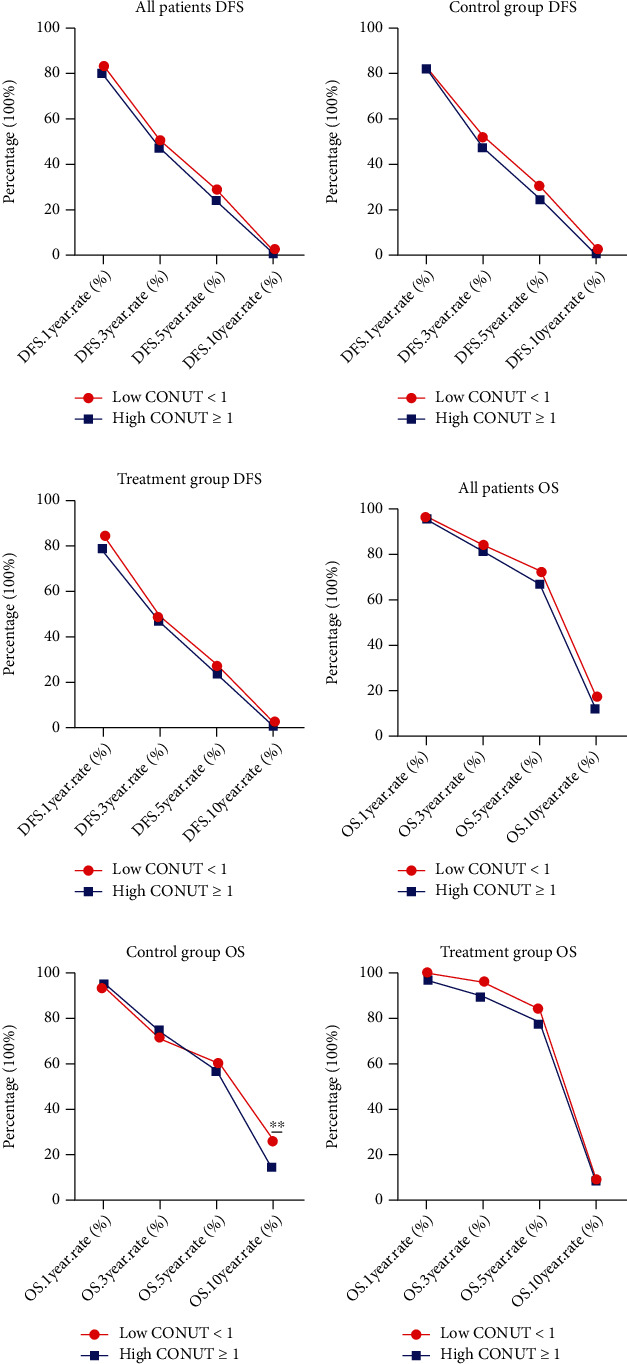
The 1-year, 3-year, 5-year, and 10-year rates of DFS and OS in breast cancer patients. (a) The 1-year, 3-year, 5-year, and 10-year rates of DFS of all patients with breast cancer. (b) The 1-year, 3-year, 5-year, and 10-year rates of OS of all patients with breast cancer. (c) The 1-year, 3-year, 5-year, and 10-year rates of DFS of patients with breast cancer in the control group. (d) The 1-year, 3-year, 5-year, and 10-year rates of OS of patients with breast cancer in the control group. (e) The 1-year, 3-year, 5-year, and 10-year rates of DFS of patients with breast cancer in treatment group. (f) The 1-year, 3-year, 5-year, and 10-year rates of OS of patients with breast cancer in treatment group.

In this study, the 1-, 3-, 5-, and 10-year rates of DFS and OS of all individuals in the low CONUT cohort were 83.41%, 50.66%, 28.82%, 1.75%, and 96.94%, 83.84%, 72.05%, and 17.47%, respectively. The corresponding DFS and OS in the high CONUT score group were 80.26%, 47.37%, 23.68%, 0.66%, and 96.05%, 81.58%, 67.11%, and 11.18%, respectively. Although with high CONUT score had worse 1-, 3-, 5-, and 10-year rates of DFS and OS than those with low CONUT scores, yet there were no significant difference between the two groups (*p* > 0.05) (Figures 3(a) and 3(b)). In the Control group, only the 5- and 10-year OS rates of those with high CONUT scores were lower in contrast to those with lower CONUT scores, but the 1- and 3-year OS rates in the high CONUT group were higher than those in the low CONUT group. However, these differences failed to achieve statistical significance (*p* > 0.05), except in 10-year rates of OS (*p* < 0.05). Conversely, the 1-, 3-, 5-, and 10-year DFS rates of those in the high CONUT group were lower in contrast to those with lower CONUT scores (Figures 3(c) and 3(d)). In the treatment group, the 1-, 3-, 5-, and 10-year DFS and OS rates were raised in the low CONUT group in contrast to those with higher CONUT scores. However, there were no significant difference between the two groups (*p* > 0.05) (Figures 3(e) and 3(f)).

### 3.8. Calibration Curve and Decision Curve Analysis for Predicting Clinical Utility

**Figure 4:**
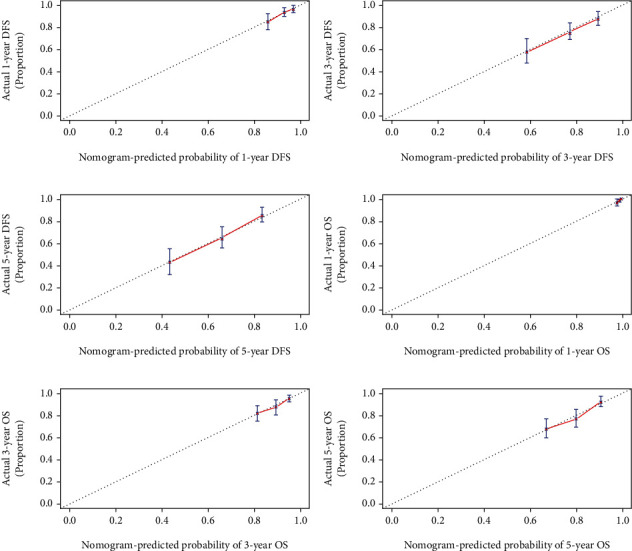
Calibration curve for evaluating the 1-, 3-, and 5-year DFS and OS rates. (a) Calibration curve for evaluating 1-year DFS rate; (b) calibration curve for evaluating 1-year OS rate; (c) calibration curve for evaluating 3-year DFS rate; (d) calibration curve for evaluating 3-year OS rate; (e) calibration curve for evaluating 5-year DFS rate; (f) calibration curve for evaluating 5-year OS rate.

**Figure 5:**
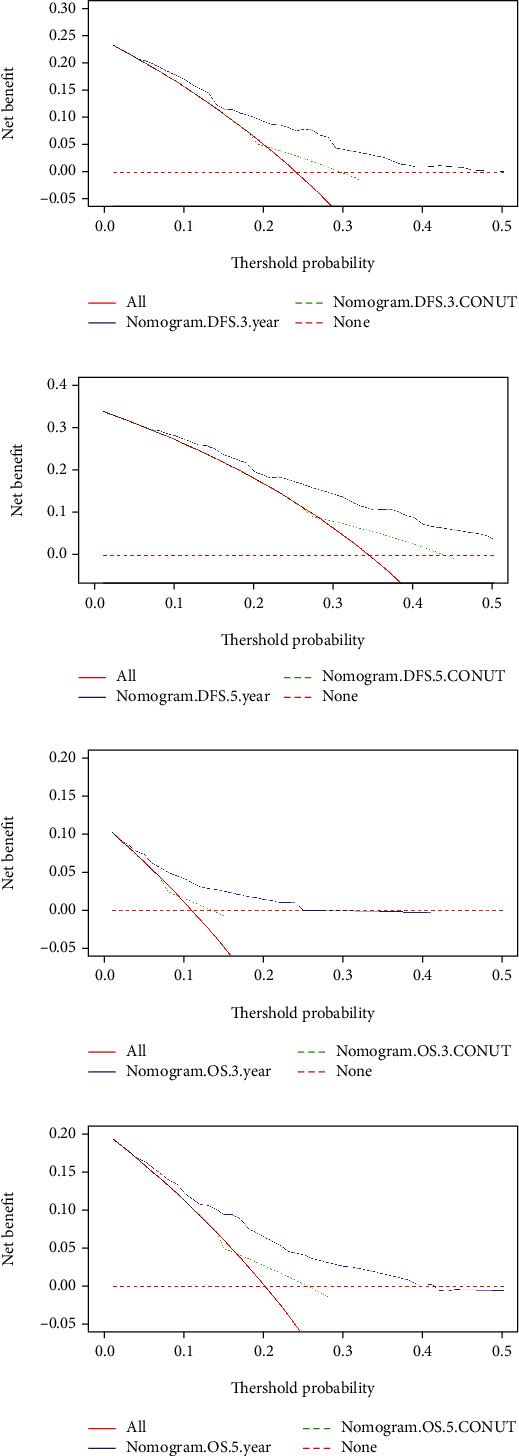
Decision curve analysis for the nomogram and only CONUT. (a) Decision curve analysis for 3-year DFS; (b) decision curve analysis for 3-year OS; (c) decision curve analysis for 5-year DFS; (d) decision curve analysis for 5-year OS.

The calibration curve was performed to predict the performance for DFS and OS after curative resection. In this study, the calibration curve shown good agreement between predicted and the actual probability at different survival time points, including 1-, 3-, and 5-year DFS and OS (Figure 4). Moreover, we also used the decision curve analysis (DCA) to test predictive clinical utility, and the result shows that compared to only CONUT, the constructed nomogram model (the nomogram incorporating the potential independent prognostic factors of OS and DFS by the multivariate analysis) yielded the best net benefit across in the range of threshold probability for 3-, 5-year DFS and OS. Furthermore, the nomogram predictive clinical utility for clinical decision-making was better than only CONUT (Figure 5).

## 4. Discussion

BC cancer prognosis appears to be closely related to the degree of systemic inflammation and nutritional status [29, 30]. Although the use of CONUT was previously confined to assessing nutritional status only, more and more evidence has highlighted its ability to predict cancer patient survival [31–33]. The various components of the CONUT score–TLC, ALB, and TC, reflect impaired immune defenses, protein reserves, and caloric depletion, respectively.

Ignacio de Ulíbarri et al. were the first to characterize the CONUT score in 2005 as a means of assessing patients' nutritional and immune status [20]. The CONUT score has been useful in several chronic diseases, including end-stage liver disease, congestive heart failure, and cancer [34–36]. A study by Shiihara et al. demonstrates that the CONUT score may be used to predict the risk of postoperative complications in pancreatic cancer patients planned for surgery, with a higher CONIT score correlating to increased risks of postoperative complications and shorter OS as well as relapse-free survival [37]. A study by Hirahara et al. linked the pTNM stage and CONUT score together as complementary parameters that predicted esophageal cancer patient survival [38]. Another investigation also indicated that the CONUT score provided invaluable prognostic information regarding stage II-III gastric cancer patients planned for curative resection and adjuvant chemotherapy and may be useful in guiding the selection of adequate preoperative nutritional interventions [39]. What is more, there are some studies predicting the similar results between CONUT score and breast cancer. A study by Huang et al. pointed out that patients in the high-CONUT score group had shorter OS and recurrence-free survival (RFS) in comparison with those in the low-CONUT score group in surgically treated breast cancer patients [40]. But they only focused on preoperative health status and failed to renew data during the whole process. Another study by Li et al. told us that breast cancer patients with high CONUT predicted the shorter DFS and OS [41]. However, the study only focused on breast cancer patients who did not receive chemotherapy before the surgery.

Our study demonstrated that a higher CONUT score was strongly linked to several clinical characteristics (weight and tumor size) and blood parameters (TC, CA153, white blood cell, hemoglobin, lymphocyte, monocyte, eosinophils, basophils, platelet, MLR, PLR, SIRI, SII, and PNI) of all enrolled patients. We also analyzed the relationship between the CONUT score and other pathological variables and found that the molecular subtype, Ki-67 status, ER status, PR status, and TOP2A status were remarkably worse in those with elevated CONUT scores.

Univariate and multivariate Cox proportional hazards regression model analyses both concurred that the CONUT score functioned as an independent prognosticator of OS and DFS. Individuals possessing lower CONUT scores demonstrated longer mean DFS and OS in contrast to those with higher scores. Moreover, we also successfully constructed a nomogram as a visual tool that is useful in predicting BC patient prognosis according to the multivariate Cox proportional hazard regression analysis on DFS and OS. Moreover, the calibration curve shown a good agreement between predicted and the actual probability at different survival time points, including 1-, 3-, and 5-year DFS and OS rates. Furthermore, the DCA was used to quantify the net benefits at different threshold probabilities, and nomograms predictive clinical utility for clinical decision-making was better than only CONUT. These results lay the foundation for the use of novel immune-nutrition evaluation tools in guiding postoperative BC patient management.

The biological mechanism of CONUT as a prognostic indicator of BC has not been clearly explained. We seek to provide a brief explanation of the relevance of each component of this score. Cholesterol is metabolized by the liver and represents a key cell membrane molecule that actively takes part in cellular metabolism [42, 43]. Gao et al. found that low circulating cholesterol concentration was associated with enhanced cancer morbidity and mortality and appeared to be a good prognostic marker in diffuse large B-cell lymphoma (DLBCL) [44]. Other investigations suggest that lower cholesterol levels may alter immune cell membranes, thus limiting their immune function and ultimately translating to poorer cancer patient prognosis [45]. Serum albumin level is widely used as a marker of nutritional and immunological status, with lower levels reflecting poorer nutrition and immune states [46, 47]. One study by Fujii et al. links low serum albumin levels to poorer BC prognosis [48]. Total lymphocyte CONUT, on the other hand, is a critical mediator of cell-mediated immunity. Lymphocytes, which can be divided into T lymphocytes (T-cells), B lymphocytes (B-cells), and natural killer (NK) cells, and played an essential role in mediating response to the proliferation, invasion, and metastasis of cancer cells [49]. A low total lymphocyte CONUT reflects suboptimal immune status and is an independent factor of poor cancer patient prognosis [50]. All CONUT score components relate to an individual's nutritional status and immune capacity, with each parameter exerting important influence over tumor initiation, occurrence, and progression.

Several limitations in this study require consideration upon the interpretation of study results. Firstly, this analysis is a retrospective study and has a relatively small sample size. Thus, further studies with prospective and multicenter studies are needed. Secondly, selection bias cannot be underestimated, and further prospective study with a larger sample size is imperative to validate this study's results. Thirdly, it is important to consider that the cut-off CONUT score value determined from this study was based on our patient cohort and may not be applicable in other cohorts.

The CONUT represents a novel independent prognostic factor in BC patients that may aid in predicting survival rates. The CONUT score is an easily replicable, convenient, and accessible parameter that can be used in daily practice.

## Figures and Tables

**Table 1:** Definition of CONUT score.

Parameter	Malnutritional state			
	None	Light	Moderate	Severe
ALB (g/L)	≥35.0	30.0-34.9	25.0-29.9	<25.0
Score	0	2	4	6
TLC (×10^9^/L)	≥1.6	1.2-1.59	0.8-1.19	<0.8
Score	0	1	2	3
TC (mmol/L)	≥10	7.78-9.99	5.56-7.77	<5.56
Score	0	1	2	3

Dysnutritional score	Normal	Light	Moderate	Severe
Total CONUT score	0-1	2-4	5-8	9-12

^#^ALB: serum albumin concentration, TLC: total peripheral lymphocyte CONUT, TC: total serum cholesterol. CONUT score = serum albumin concentration score + total peripheral lymphocyte CONUT score + total serum cholesterol score.

**Table 2:** Patients' baseline data.

Parameters	All patients (*n* = 381)		*χ* ^2^	*p* value	Control group (*n* = 187)		*χ* ^2^	*p* value	Treatment group (194)		*χ* ^2^	*p* value
Cases (*n*)	LOW 229	HIGH 152			Low 111	High 76			Low 118	High 76		
Age (years)			1.372	0.241			1.503	0.220			0.251	0.617
< 50	108(57.14%)	81(42.86%)			44(54.32%)	37(45.68%)			64(59.26%)	44(40.74%)		
≥ 50	121(63.02%)	71(36.98%)			67(63.21%)	39(36.79%)			54(62.79%)	32(37.21%)		
Marital status			1.975	0.577							2.712	0.438
Married	214(59.78%)	144(40.22%)			110(60.11%)	73(39.89%)			104(59.43%)	71(40.57%)		
Unmarried	10(76.92%)	3(23.08%)			1(50.00%)	1(50.00%)			9(81.82%)	2(18.18%)		
Divorced	2(50.00%)	2(50.00%)			0(0.00%)	0(0.00%)			2(50.00%)	2(50.00%)		
Widowed	3(50.00%)	3(50.00%)			0(0.00%)	2(100.00%)			3(75.00%)	1(25.00%)		
Occupation			1.560	0.456			2.195	0.334			1.821	0.401
Mental worker	82(56.94%)	62(43.06%)			26(50.98%)	25(49.02%)			56(60.22%)	37(39.78%)		
Manual worker	53(58.89%)	37(41.11%)			37(62.71%)	22(37.29%)			16(51.61%)	15(48.39%)		
Others	94(63.95%)	53(36.05%)			48(62.34%)	29(37.66%)			46(65.71%)	24(34.29%)		
Weight (kg)			10.161	0.001			11.232	0.001			1.342	0.247
< 62	90(51.43%)	85(48.57%)			41(46.59%)	47(53.41%)			49(56.32%)	38(43.68%)		
≥ 62	139(67.48%)	67(32.52%)			70(70.71%)	29(29.29%)			69(64.49%)	38(35.51%)		
Height (m)			0.263	0.608			1.136	0.286			0.094	0.760
< 1.58	101(61.59%)	63(38.41%)			57(63.33%)	33(36.67%)			44(59.46%)	30(40.54%)		
≥ 1.58	128(58.99%)	89(41.01%)			54(55.67%)	43(44.33%)			74(61.67%)	46(38.33%)		
BMI			2.270	0.132			3.020	0.082			0.178	0.673
< 24.45	107(56.32%)	83(43.68%)			47(52.81%)	42(47.19%)			60(59.41%)	41(40.59%)		
≥ 24.45	122(63.87%)	69(36.13%)			64(65.31%)	34(34.69%)			58(62.37%)	35(37.63%)		
Family history			0.047	0.829			0.011	0.916			0.044	0.835
No	171(60.42%)	112(39.58%)			84(59.57%)	57(40.43%)			87(61.27%)	55(38.73%)		
Yes	58(59.18%)	40(40.82%)			27(58.70%)	19(41.30%)			31(59.62%)	21(40.38%)		
Menarche age (year)			0.062	0.802			0.211	0.646			0.002	0.960
< 14	80(59.26%)	55(40.74%)			33(56.90%)	25(43.10%)			47(61.04%)	30(38.96%)		
≥ 14	149(60.57%)	97(39.43%)			78(60.47%)	51(39.53%)			71(60.68%)	46(39.32%)		
Menopause			1.419	0.233			0.883	0.347			0.588	0.443
No	126(57.53%)	93(42.47%)			58(56.31%)	45(43.69%)			68(58.62%)	48(41.38%)		
Yes	103(63.58%)	59(36.42%)			53(63.10%)	31(36.90%)			50(64.10%)	28(35.90%)		
ABO blood type			0.962	0.811			3.076	0.381			1.815	0.612
A	55(61.80%)	34(38.20%)			28(66.67%)	14(33.33%)			27(57.45%)	20(42.55%)		
B	71(61.74%)	44(38.26%)			36(64.29%)	20(35.71%)			35(59.32%)	24(40.68%)		
O	81(59.56%)	55(40.44%)			38(52.78%)	34(47.22%)			43(67.19%)	21(32.81%)		
AB	22(53.66%)	19(46.34%)			9(52.94%)	8(47.06%)			13(54.17%)	11(45.83%)		
Tumor site			0.636	0.801			0.086	0.769			0.004	0.949
Right	113(60.75%)	73(39.25%)			55(60.44%)	36(39.56%)			58(61.05%)	37(38.95%)		
Left	116(59.49%)	79(40.51%)			56(58.33%)	40(41.67%)			60(60.61%)	39(39.39%)		
Type of surgery			0.068	0.795			0.001	0.966			0.153	0.696
Mastectomy	214(60.28%)	141(39.72%)			105(59.32%)	72(40.68%)			109(61.24%)	69(38.76%)		
Breast-conserving surgery	15(57.69%)	11(42.31%)			6(60.00%)	4(40.00%)			9(56.25%)	7(43.75%)		
Tumor size			6.368	0.041			1.719	0.424			6.800	0.033
≤2 cm	94(61.04%)	60(38.96%)			33(63.46%)	19(36.54%)			61(59.80%)	41(40.20%)		
>2 and< 5 cm	114(63.69%)	65(36.31%)			66(60.00%)	44(40.00%)			48(69.57%)	21(30.43%)		
≥ 5 cm	21(43.75%)	27(56.25%)			12(48.00%)	13(52.00%)			9(39.13%)	14(60.87%)		
Postoperative complications			0.104	0.748			0.057	0.812			0.041	0.839
No	220(60.27%)	145(39.73%)			106(59.55%)	72(40.45%)			114(60.96%)	73(39.04%)		
Yes	9(56.25%)	7(43.75%)			5(55.56%)	4(44.44%)			4(57.14%)	3(42.86%)		
Postoperative chemotherapy			1.405	0.236			1.738	0.187			0.168	0.682
No	89(64.03%)	50(35.97%)			28(68.29%)	13(31.71%)			61(62.24%)	37(37.76%)		
Yes	140(57.85%)	102(42.15%)			83(56.85%)	63(43.15%)			57(59.38%)	39(40.63%)		
Postoperative radiotherapy			0.153	0.695			2.635	0.105			1.446	0.229
No	49(62.03%)	30(37.97%)			32(69.57%)	14(30.43%)			17(51.52%)	16(48.48%)		
Yes	180(59.60%)	122(40.40%)			79(56.03%)	62(43.97%)			101(62.73%)	60(37.27%)		
Postoperative endocrine therapy			0.781	0.377			0.066	0.797			0.945	0.331
No	92(57.50%)	68(42.50%)			49(58.33%)	35(41.67%)			43(56.58%)	33(43.42%)		
Yes	137(61.99%)	84(38.01%)			62(60.19%)	41(39.81%)			75(63.56%)	43(36.44%)		
Postoperative targeted therapy			1.306	0.253			2.529	0.112			0.364	0.546
No	190(61.49%)	119(38.51%)			106(60.92%)	68(39.08%)			84(62.22%)	51(37.78%)		
Yes	39(54.17%)	33(45.83%)			5(38.46%)	8(61.54%)			34(57.63%)	25(42.37%)		

^#^BMI: body mass index.

**Table 3:** The relationships between the CONUT score and blood parameters of BC patients.

Parameters	All patients (*n* = 381)		*χ* ^2^	*p* value	Control group (*n* = 187)		*χ* ^2^	*p* value	Treatment group (*n* = 194)		*χ* ^2^	*p* value
Cases (*n*)	LOW 229	HIGH 152			Low 111	High 76			Low 118	High 76		
ALB (g/L)			0.252	0.615			0.266	0.606			0.081	0.776
< 44.10	116(61.38%)	73(38.62%)			67(60.91%)	43(39.09%)			49(62.03%)	30(37.97%)		
≥ 44.10	113(58.85%)	79(41.15%)			44(57.14%)	33(42.86%)			69(60.00%)	46(40.00%)		
TC (mmol/L)			4.555	0.033			0.996	0.318			3.946	0.047
< 4.38	104(54.74%)	86(45.26%)			59(56.19%)	46(43.81%)			45(52.94%)	40(47.06%)		
≥ 4.38	125(65.45%)	66(34.55%)			52(63.41%)	30(36.59%)			73(66.97%)	36(33.03%)		
TP(g/L)			0.044	0.837			0.200	0.655			0.008	0.927
< 71.90	112(59.57%)	76(40.43%)			46(57.50%)	34(42.50%)			66(61.11%)	42(38.89%)		
≥ 71.90	117(60.62%)	76(39.38%)			65(60.75%)	42(39.25%)			52(60.47%)	34(39.53%)		
PALB (mg/dl)			1.276	0.259			2.834	0.092			0.006	0.939
< 24	101(57.06%)	76(42.94%)			46(52.87%)	41(47.13%)			55(61.11%)	35(38.89%)		
≥ 24	128(62.75%)	76(36.32%)			65(65.00%)	35(35.00%)			63(60.58%)	41(43.62%)		
CA125 (U/mL)			2.025	0.155			0.857	0.355			1.345	0.246
< 13.70	121(63.68%)	69(36.32%)			66(62.26%)	40(37.74%)			55(65.48%)	29(34.52%)		
≥ 13.70	108(56.54%)	83(43.46%)			45(55.56%)	36(44.44%)			63(57.27%)	47(42.73%)		
CA153 (U/mL)			4.205	0.041			1.967	0.161			2.467	0.116
< 12.69	124(65.26%)	66(34.74%)			67(63.81%)	38(36.19%)			57(67.06%)	28(32.94%)		
≥ 12.69	105(54.97%)	86(45.03%)			44(53.66%)	38(46.34%)			61(55.96%)	48(44.04%)		
CEA (ng/mL)			0.002	0.097			0.171	0.679			0.224	0.636
< 1.86	114(60.00%)	76(40.00%)			56(60.87%)	36(39.13%)			58(59.18%)	40(40.82%)		
≥ 1.86	115(60.21%)	76(39.79%)			55(57.89%)	40(42.11%)			60(62.50%)	36(37.50%)		
Before chemotherapy												
White blood cell (W) (×10^9^/L)			32.391	<0.0001			7.831	0.005			27.533	<0.0001
< 5.92	87(45.79%)	103(54.21%)			53(50.48%)	52(49.52%)			34(40.00%)	51(60.00%)		
≥ 5.92	142(74.35%)	49(25.65%)			58(70.73%)	24(29.27%)			84(77.06%)	25(22.94%)		
Hemoglobin (Hb) (×10^9^/L)			4.589	0.032			3.433	0.064			1.510	0.219
< 131	92(54.12%)	78(45.88%)			39(51.32%)	37(48.68%)			53(56.38%)	41(43.62%)		
≥ 131	137(64.93%)	74(35.07%)			72(64.86%)	39(35.14%)			65(65.00%)	35(35.00%)		
Neutrophil (N) (×10^9^/L)			2.528	0.112			0.072	0.788			3.736	0.053
< 3.41	106(56.08%)	83(43.92%)			65(58.56%)	46(41.44%)			41(52.56%)	37(47.44%)		
≥ 3.41	123(64.06%)	69(35.94%)			46(60.53%)	30(39.47%)			77(66.38%)	39(33.62%)		
Lymphocyte (TLC) (×10^9^/L)			107.713	<0.0001			34.847	<0.0001			79.147	<0.0001
< 1.87	64(33.86%)	125(66.14%)			29(35.37%)	53(64.63%)			35(32.71%)	72(67.29%)		
≥ 1.87	165(85.94%)	27(14.06%)			82(78.10%)	23(21.90%)			83(95.40%)	4(4.60%)		
Monocyte (M) (×10^9^/L)			12.091	0.001			3.227	0.072			10.506	0.001
< 0.34	91(50.84%)	88(49.16%)			63(54.31%)	53(45.69%)			28(44.44%)	35(55.56%)		
≥ 0.34	138(68.32%)	64(31.68%)			48(67.61%)	23(32.39%)			90(68.70%)	41(31.30%)		
Eosinophils (E) (×10^9^/L)			5.890	0.015			1.635	0.201			4.876	0.027
< 0.08	102(53.97%)	87(46.03%)			45(54.22%)	38(45.78%)			57(53.77%)	49(46.23%)		
≥ 0.08	127(66.15%)	65(33.85%)			66(63.46%)	38(36.54%)			61(69.32%)	27(30.68%)		
Basophils (B) (×10^9^/L)			4.042	0.044			0.200	0.655			5.751	0.016
< 0.02	79(53.74%)	68(46.26%)			46(57.50%)	34(42.50%)			33(49.25%)	34(50.75%)		
≥ 0.02	150(64.10%)	84(35.90%)			65(60.75%)	42(39.25%)			85(66.93%)	42(33.07%)		
Platelet (P) (×10^9^/L)			4.555	0.033			2.014	0.156			2.469	0.116
< 235	104(54.74%)	86(45.26%)			60(55.05%)	49(44.95%)			44(54.32%)	37(45.68%)		
≥ 235	125(65.45%)	66(34.55%)			51(65.38%)	27(34.62%)			74(65.49%)	39(34.51%)		
NLR			0.567	0.451			2.040	0.153			0.128	0.720
< 1.82	110(58.20%)	79(41.80%)			51(54.26%)	43(45.74%)			59(62.11%)	36(37.89%)		
≥ 1.82	119(61.98%)	73(38.02%)			60(64.52%)	33(35.48%)			59(59.60%)	40(40.40%)		
MLR			21.973	<0.0001			6.701	0.010			19.511	<0.0001
< 0.19	136(71.96%)	53(28.04%)			79(66.39%)	40(33.61%)			57(81.43%)	13(18.57%)		
≥ 0.19	93(48.44%)	99(51.56%)			32(46.38%)	36(52.17%)			61(49.59%)	63(51.22%)		
PLR			39.931	<0.0001			13.612	<0.0001			31.881	<0.0001
< 126.50	145(75.92%)	46(24.08%)			82(69.49%)	36(30.51%)			63(86.30%)	10(13.70%)		
≥ 126.50	84(44.21%)	106(55.79%)			29(42.03%)	40(57.97%)			55(45.45%)	66(54.55%)		
SIRI			4.562	0.033			1.917	0.166			3.934	0.047
< 0.64	122(65.59%)	64(34.41%)			78(62.90%)	46(37.10%)			44(70.97%)	18(29.03%)		
≥ 0.64	107(54.87%)	88(45.13%)			33(52.38%)	30(47.62%)			74(56.06%)	58(43.94%)		
SII			11.490	0.001			5.400	0.020			7.409	0.006
< 434.20	131(68.59%)	60(31.41%)			77(65.81%)	40(34.19%)			54(72.97%)	20(27.03%)		
≥ 434.20	98(51.58%)	92(48.42%)			34(48.57%)	36(51.43%)			64(53.33%)	56(46.67%)		
PNI			38.360	<0.0001			10.310	0.001			30.555	<0.0001
< 53.50	84(44.44%)	105(55.56%)			48(48.48%)	51(51.52%)			36(40.00%)	54(60.00%)		
≥ 53.50	145(75.52%)	47(24.48%)			63(71.59%)	25(28.41%)			82(78.85%)	22(21.15%)		

^#^ALB: serum albumin concentration; TC: total serum cholesterol; TP: total protein; PALB: prealbumin; NLR = Neutrophil/Lymphocyte ratio; MLR = Monocyte/Lymphocyte ratio; PLR = Platelet/Lymphocyte ratio; SIRI (Systemic inflammation response index) = neutrophil (N) × monocyte (M)/lymphocyte (TLC); SII (Systemic Immune–inflammation Index) = (neutrophil (N) × platelet (P))/lymphocyte (TLC); PNI (Prognostic nutritional index) = ALB + 5 × total lymphocyte CONUT (TLC).

**Table 4:** The relationships between the CONUT score and pathological variables of BC patients.

Parameters	All patients (*n*=381)		*χ* ^2^	*p* value	Control group (*n*=187)		*χ* ^2^	*p* value	Treatment group (*n*=194)		*χ* ^s^	*p* value
Cases (*n*)	LOW 229	HIGH 152			Low 111	High 76			Low 118	High 76		
Postoperative pathology (IHC)												
Molecular subtype			9.941	0.041			5.068	0.280			12.004	0.017
Luminal A	17(58.62%)	12(41.38%)			10(76.92%)	3(23.08%)			7(43.75%)	9(56.25%)		
Luminal B HER2+	19(48.72%)	20(51.28%)			10(55.56%)	8(44.44%)			9(42.86%)	12(57.14%)		
Luminal B HER2-	119(68.39%)	55(31.61%)			60(63.83%)	34(36.17%)			59(73.75%)	21(26.25%)		
HER2 enriched	36(54.55%)	30(45.45%)			14(46.67%)	16(53.33%)			22(61.11%)	14(38.89%)		
Triple negative	38(52.05%)	35(47.95%)			17(53.13%)	15(46.88%)			21(51.22%)	20(48.78%)		
ER status			10.393	0.016			9.187	0.027			4.253	0.235
0-25%	89(51.74%)	83(48.26%)			42(49.41%)	43(50.59%)			47(54.02%)	40(45.98%)		
26-50%	35(71.43%)	14(28.57%)			26(78.79%)	7(21.21%)			9(56.25%)	7(43.75%)		
51-75%	19(73.08%)	7(26.92%)			7(70.00%)	3(30.00%)			12(75.00%)	4(25.00%)		
76-100%	86(64.18%)	48(35.82%)			36(61.02%)	23(38.98%)			50(66.67%)	25(33.33%)		
PR status			9.655	0.022			8.214	0.042			3.681	0.298
0-25%	129(55.13%)	105(44.87%)			49(50.52%)	48(49.48%)			80(58.39%)	57(41.61%)		
26-50%	47(75.81%)	15(24.19%)			27(75.00%)	9(25.00%)			20(76.92%)	6(23.08%)		
51-75%	16(69.57%)	7(30.43%)			7(77.78%)	2(22.22%)			9(64.29%)	5(35.71%)		
76-100%	37(59.68%)	25(40.32%)			28(62.22%)	17(37.78%)			9(52.94%)	8(47.06%)		
HER2 status			2.160	0.141			2.344	0.126			0.364	0.546
Negative (0^--++)^	171(62.41%)	103(37.59%)			87(62.59%)	52(37.41%)			84(62.22%)	51(37.78%)		
Positive (+++)	58(54.21%)	49(45.79%)			24(50.00%)	24(50.00%)			34(57.63%)	25(42.37%)		
Ki-67 status			8.813	0.032			6.221	0.101			4.863	0.182
0-25%	148(66.37%)	75(33.63%)			85(64.89%)	46(35.11%)			63(68.48%)	29(31.52%)		
26-50%	52(51.49%)	49(48.51%)			18(43.90%)	23(56.10%)			34(56.67%)	26(43.33%)		
51-75%	18(51.43%)	17(48.57%)			6(50.00%)	6(50.00%)			12(52.17%)	11(47.83%)		
76-100%	11(50.00%)	11(50.00%)			2(66.67%)	1(33.33%)			9(47.37%)	10(52.63%)		
AR status			2.901	0.407							2.855	0.414
0-25%	221(59.73%)	149(40.27%)			111(59.36%)	76(40.64%)			110(60.11%)	73(39.89%)		
26-50%	2(66.67%)	1(33.33%)			0(0.00%)	0(0.00%)			2(66.67%)	1(33.33%)		
51-75%	4(100.00%)	0(0.00%)			0(0.00%)	0(0.00%)			4(100.00%)	0(0.00%)		
76-100%	2(50.00%)	2(50.00%)			0(0.00%)	0(0.00%)			2(50.00%)	2(50.00%)		
P53 status			7.605	0.055			11.823	0.008			7.181	0.066
0-25%	179(64.16%)	100(35.84%)			91(63.19%)	53(36.81%)			88(65.19%)	47(34.81%)		
26-50%	18(51.43%)	17(48.57%)			7(70.00%)	3(30.00%)			11(44.00%)	14(56.00%)		
51-75%	29(46.77%)	33(53.23%)			10(33.33%)	20(66.67%)			19(59.38%)	13(40.63%)		
76-100%	3(60.00%)	2(40.00%)			3(100.00%)	0(0.00%)			0(0.00%)	2(100.00%)		
TOP2A status			10.133	0.017			6.524	0.038			6.182	0.103
0-25%	187(63.39%)	108(36.61%)			96(63.58%)	55(36.42%)			91(63.19%)	53(36.81%)		
26-50%	38(52.05%)	35(47.95%)			15(42.86%)	20(57.14%)			23(60.53%)	15(39.47%)		
51-75%	3(25.00%)	9(75.00%)			0(0.00%)	1(100.00%)			3(27.27%)	8(72.73%)		
76-100%	1(100.00%)	0(0.00%)			0(0.00%)	0(0.00%)			1(100.00%)	0(0.00%)		
CK5/6 status			1.693	0.193			0.001	0.976			2.395	0.122
Negative	211(61.16%)	134(38.84%)			108(59.34%)	74(40.66%)			103(63.19%)	60(36.81%)		
Positive	18(50.00%)	18(50.00%)			3(60.00%)	2(40.00%)			15(48.39%)	16(51.61%)		
E-cad status			0.042	0.838			2.183	0.140			0.294	0.588
Negative	138(60.53%)	90(39.47%)			99(61.49%)	62(38.51%)			39(58.21%)	28(41.79%)		
Positive	91(59.48%)	62(40.52%)			12(46.15%)	14(53.85%)			79(62.20%)	48(37.80%)		
EGFR status			3.092	0.079			0.303	0.582			3.738	0.053
Negative	194(62.18%)	118(37.82%)			107(59.78%)	72(40.22%)			87(65.41%)	46(34.59%)		
Positive	35(50.72%)	34(49.28%)			4(50.00%)	4(50.00%)			31(50.82%)	30(49.18%)		
Lymph vessel invasion			0.712	0.399			1.907	0.167			0.312	0.860
Negative	144(58.54%)	102(41.46%)			68(55.74%)	54(44.26%)			76(61.29%)	48(38.71%)		
Positive	85(62.96%)	50(37.04%)			43(66.15%)	22(33.85%)			42(60.00%)	28(40.00%)		
Neural invasion			0.340	0.560			0.001	0.976			0.582	0.445
Negative	202(60.66%)	131(39.34%)			108(59.34%)	74(40.66%)			94(62.25%)	57(37.75%)		
Positive	27(56.25%)	21(43.75%)			3(60.00%)	2(40.00%)			24(55.81%)	19(44.19%)		

^#^ER: estrogen receptor; PR: progesterone receptor; HER2: human epidermal growth factor receptor; AR: androgen receptor, E-cad: e-cadnerin; EGFR: epithelial growth factor receptor.

**Table 5:** Univariate and multivariate Cox regression survival analyses of the CONUT score for the prediction of DFS and OS in BC patients.

Parameters	DFS				OS			
	Univariate analysis	*p* value	Multivariate analysis	*p* value	Univariate analysis	*p* value	Multivariate analysis	*p* value
	Hazard ratio (95% CI)		Hazard ratio (95% CI)		Hazard ratio (95% CI)		Hazard ratio (95% CI)	
Cases (*n*)								
Age (years)		0.183				0.646		
< 50	1 (reference)				1 (reference)			
≥ 50	1.502(0.825-2.733)				1.195(0.558-2.558)			
BMI		0.595				0.778		
< 24.45	1 (reference)				1 (reference)			
≥ 24.45	1.281(0.514-3.196)				0.899(0.428-1.888)			
Family history		0.229				0.857		
No	1 (reference)				1 (reference)			
Yes	0.694(0.383-1.258)				0.952(0.555-1.633)			
Menopause		0.312				0.943		
No	1 (reference)				1 (reference)			
Yes	0.660(0.295-1.477)				1.027(0.494-2.134)			
Type of surgery		0.608				0.931		
Mastectomy	1 (reference)				1 (reference)			
Breast-conserving surgery	0.685(0.162-2.908)				0.959(0.376-2.450)			
Tumor size		0.061				0.918		
≤2 cm	1 (reference)				1 (reference)			
> 2 and< 5 cm	2.093(1.134-3.863)	0.018			1.093(0.665-1.798)	0.726		
≥ 5 cm	1.424(0.639-3.173)	0.388			1.133(0.524-2.450)	0.752		
Postoperative chemotherapy		<0.0001		<0.0001		<0.0001		<0.0001
No	1 (reference)		1 (reference)		1 (reference)		1 (reference)	
Yes	3.865(1.935-7.722)		4.104(2.312-7.285)		3.256(1.937-5.473)		3.594(1.953-6.613)	
Postoperative radiotherapy		<0.0001		<0.0001		0.009		0.010
No	1 (reference)		1 (reference)		1 (reference)		1 (reference)	
Yes	0.217(0.102-0.461)		0.348(0.196-0.618)		0.474(0.271-0.828)		0.451(0.246-0.828)	
Postoperative endocrine therapy		0.204				0.693		
No	1 (reference)				1 (reference)			
Yes	0.637(0.318-1.276)				0.878(0.460-1.676)			
Postoperative targeted therapy		0.301				0.884		
No	1 (reference)				1 (reference)			
Yes	1.560(0.672-3.622)				0.946(0.450-1.990)			
ALB (g/L)		0.984				0.922		
< 44.10	1 (reference)				1 (reference)			
≥ 44.10	1.007(0.509-1.990)				0.969(0.512-1.832)			
Total serum cholesterol (TC) (mmol/L)		0.015		0.012		0.021		0.002
< 4.38	1 (reference)		1 (reference)		1 (reference)		1 (reference)	
≥ 4.38	0.488(0.274-0.871)		0.591(0.392-0.893)		0.638(0.436-0.933)		0.484(0.308-0.760)	
TP(g/L)		0.274				1.000		
< 71.90	1 (reference)				1 (reference)			
≥ 71.90	0.712(0.388-1.308)				1.000(0.600-1.666)			
PALB(mg/dl)		0.055				0.745		
< 24	1 (reference)				1 (reference)			
≥ 24	0.577(0.329-1.012)				0.921(0.562-1.510)			
White blood cell (W) (×10^9^/L)		0.360				0.875		
< 5.92	1 (reference)				1 (reference)			
≥ 5.92	1.483(0.638-3.446)				1.060(0.517-2.173)			
Neutrophil (N) (×10^9^/L)		0.195				0.937		
< 3.41	1 (reference)				1 (reference)			
≥ 3.41	0.582(0.257-1.319)				0.971(0.467-2.016)			
Total lymphocyte (TLC) (×10^9^/L)		0.670				0.739		
< 1.87	1 (reference)				1 (reference)			
≥ 1.87	1.192(0.531-2.675)				1.122(0.570-2.209)			
Monocyte (M) (×10^9^/L)		0.399				0.910		
< 0.34	1 (reference)				1 (reference)			
≥ 0.34	1.402(0.639-3.075)				0.963(0.503-1.844)			
Platelet (P) (×10^9^/L)		0.315				0.706		
< 235	1 (reference)				1 (reference)			
≥ 235	1.379(0.736-2.582)				0.901(0.522-1.553)			
CONUT		<0.0001		<0.0001		<0.0001		<0.0001
< 1	1 (reference)		1 (reference)		1 (reference)		1 (reference)	
≥ 1	3.184(1.786-5.677)		2.465(1.642-3.700)		2.326(1.578-3.429)		2.775(1.791-4.300)	
Molecular subtype		0.145				0.955		
Luminal A	1 (reference)				1 (reference)			
Luminal B HER2+	0.419(0.098-1.795)	0.241			0.668(0.122-3.648)	0.641		
Luminal B HER2-	1.650(0.801-3.400)	0.175			1.092(0.459-2.598)	0.843		
HER2 enriched	0.314(0.066-1.484)	0.144			0.809(0.144-4.547)	0.810		
Triple negative	1.076(0.374-3.094)	0.892			1.424(0.393-5.166)	0.591		
ER status		0.130				0.073		
0-25%	1 (reference)				1 (reference)			
26-50%	1.260(0.561-2.828)	0.575			1.690(0.740-3.858)	0.213		
51-75%	1.507(0.574-3.956)	0.405			3.353(1.347-8.344)	0.009		
76-100%	0.703(0.345-1.432)	0.332)			1.594(0.768-3.307)	0.211		
PR status		0.458				0.995		
0-25%	1 (reference)				1 (reference)			
26-50%	0.761(0.344-1.683)	0.501			1.104(0.518-2.356)	0.798		
51-75%	2.332(0.602-9.030)	0.220			1.028(0.364-2.907)	0.958		
76-100%	0.858(0.374-1.970)	0.719			1.060(0.522-2.154)	0.872		
HER2 status		0.089				0.443		
Negative (0--++)	1 (reference)				1 (reference)			
Positive (+++)	2.842(0.853-9.473)				1.803(0.401-8.110)			
Ki-67 status		0.059				0.966		
0-25%	1 (reference)				1 (reference)			
26-50%	1.132(0.552-2.319)	0.736			0.967(0.516-1.811)	0.915		
51-75%	2.755(1.026-7.396)	0.044			1.090(0.469-2.535)	0.841		
76-100%	0.124(0.008-1.973)	0.139			0.751(0.190-2.964)	0.682		
AR status		0.927				0.983		
0-25%	1 (reference)				1 (reference)			
26-50%	3.019(0.467-19.517)	0.981			0.862(0.036-20.773)	0.927		
51-75%	0.000(0.000-6.410)	0.975			1.048(0.089-12.333)	0.970		
76-100%	2.123(0.240-18.756)	0.498			1.519(0.181-12.737)	0.700		
P53 status		0.655				0.934		
0-25%	1 (reference)				1 (reference)			
26-50%	1.471(0.757-2.859)	0.255			0.883(0.373-2.093)	0.778		
51-75%	0.902(0.510-1.598)	0.725			1.073(0.553-2.080)	0.836		
76-100%	0.812(0.147-4.477)	0.811			1.669(0.267-10.420)	0.583		
CK5/6 status		0.711				0.702		
Negative	1 (reference)				1 (reference)			
Positive	1.169(0.513-2.664)				1.237(0.415-3.686)			
E-cad status		0.597				0.900		
Negative	1 (reference)				1 (reference)			
Positive	1.219(0.585-2.542)				1.038(0.579-1.859)			
EGFR status		0.487				0.875		
Negative	1 (reference)				1 (reference)			
Positive	0.686(0.238-1.983)				0.936(0.411-2.133)			
Lymph vessel invasion		0.001		0.037		0.937		
Negative	1 (reference)		1 (reference)		1 (reference)			
Positive	2.691(1.469-4.930)		1.560(1.028-2.368)		1.021(0.610-1.708)			
Neural invasion		0.119				0.874		
Negative	1 (reference)				1 (reference)			
Positive	0.489(0.199-1.201)				0.941(0.447-1.983)			

## Data Availability

The data used to support the findings of this study are available from the corresponding author upon request.
